# Supplementary material for: Native European crayfish Astacus astacus competitive in staged confrontation with the invasive crayfish Faxonius limosus and Procambarus acutus
Source: PLoS One. 2022 Jan 27;17(1):e0263133. doi: 10.1371/journal.pone.0263133 (PMC8794086; doi:10.1371/journal.pone.0263133)
Supplement: S1 Table — Number of "First Attacks", "Wins" and "Retreats" observed for each trialed crayfish. (PDF) [file pone.0263133.s001.pdf]

Trial	Gender	Carapace length (mm)		First attacks		Wins		Retreats	
		F. limosus	***P. acutus***	***F. limosus***	***P. acutus***	***F. limosus***	***P. acutus***	***F. limosus***	***P. acutus***
1	Male	47.47	44.26	3	12	1	8	5	0
2	Male	39.02	39.14	1	8	0	3	5	1
3	Male	44.98	44.54	3	11	2	3	7	1
4	Male	43.15	43.7	14	15	3	5	6	8
5	Male	42.12	41.32	2	2	0	0	3	1
6	Female	42.58	42.74	2	1	2	0	3	0
7	Female	38.44	39.39	3	3	1	0	2	1
8	Female	43.72	44.87	2	9	0	1	10	1
9	Female	43.13	43.43	0	4	0	1	4	1
10	Female	44.77	45.82	7	6	2	4	2	3
11	Female	40.75	40.77	4	11	0	1	2	13
12	Male	45.23	45.39	7	4	3	0	0	13
13	Male	46.6	46.47	1	27	0	14	12	1
14	Male	40.67	40.93	1	9	0	0	10	2
15	Male	43.37	44.17	2	18	2	4	13	0
16	Male	42.79	40.99	2	15	0	4	10	4
17	Male	45.81	45.6	4	3	0	0	2	8
18	Male	45.81	46.42	2	4	0	0	8	0
