# Supplementary material for: Native European crayfish Astacus astacus competitive in staged confrontation with the invasive crayfish Faxonius limosus and Procambarus acutus
Source: PLoS One. 2022 Jan 27;17(1):e0263133. doi: 10.1371/journal.pone.0263133 (PMC8794086; doi:10.1371/journal.pone.0263133)
Supplement: S2 Table — Frequencies of shelter occupancy for male and female species pairs. (PDF) [file pone.0263133.s002.pdf]

A. astacus* vs. *F. limosus

	Occupied shelter		Occupied shelter
A. astacus male	5	*A. astacus* female	4
F. limosus male	0	*F. limosus* female	1
Neither	0	Neither	0
Total	5	Total	5

A. astacus* vs. *P. acutus

	Occupied shelter		Occupied shelter
A. astacus male	4	*A. astacus* female	5
P. acutus male	3	*P. acutus* female	0
Neither	2	Neither	1
Total	9	Total	6

***F. limosus* vs. P. acutus**

	Occupied shelter		Occupied shelter
F. limosus male	6	*F. limosus* female	3
P. acutus male	3	*P. acutus* female	2
Neither	3	Neither	1
Total	12	Total	6

Control

	Occupied shelter	Number of trials
F. limosus male	7	7
F. limosus female	6	6
P. acutus male	3	7
P. acutus female	5	5
