# Supplementary material for: Native European crayfish Astacus astacus competitive in staged confrontation with the invasive crayfish Faxonius limosus and Procambarus acutus
Source: PLoS One. 2022 Jan 27;17(1):e0263133. doi: 10.1371/journal.pone.0263133 (PMC8794086; doi:10.1371/journal.pone.0263133)
Supplement: S3 Table — Coefficients, standard errors, t-statistics and p-values of three quasipoisson distributed log linked Generalized Linear Models (GLMs) without intercept based on 43 observations for the dependent variables "First Attacks", "Wins" and "Retreats". (PDF) [file pone.0263133.s003.pdf]

Outcomes of three intercept less Generalized Linear Models (GLMs) with quasipoisson distribution and logarithmic link function based on 43 observations for the dependent variables "First Attacks", "Wins" and "Retreats"

**First Attacks**				
	Estimate	S.E.	t-value	p-value
SexFemale	**3.21**	0.18	17.5	**<0.001**
SexMale	**3.51**	0.13	27.3	**<0.001**
SpeciesFlimAast	-0.13	0.28	-0.5	0.6
SpeciesFlimPacu	**-1.05**	0.36	-2.9	**0.006**
SexMale:SpeciesFlimAast	-0.17	0.37	-0.5	0.7
SexMale:SpeciesFlimPacu	0.19	0.42	0.5	0.7
Dispersion coefficient	4.96			

**Wins**				
	Estimate	S.E.	t-value	p-value
SexFemale	**1.47**	0.44	3.4	**0.002**
SexMale	**2.29**	0.23	9.7	**<0.001**
SpeciesFlimAast	0.47	0.58	0.8	0.4
SpeciesFlimPacu	-0.77	0.78	-1	0.3
SexMale:SpeciesFlimAast	-0.8	0.73	-1.1	0.3
SexMale:SpeciesFlimPacu	-0.05	0.87	-0.06	1
Dispersion coefficient	4.97			

**Retreats**				
	Estimate	S.E.	t-value	p-value
SexFemale	**2.86**	0.19	14.9	**<0.001**
SexMale	**3.01**	0.14	20.7	**<0.001**
SpeciesFlimAast	-0.36	0.32	-1.1	0.3
SpeciesFlimPacu	-0.91	0.36	-2.6	0.01
SexMale:SpeciesFlimAast	0.06	0.42	0.1	0.9
SexMale:SpeciesFlimPacu	0.21	0.43	0.5	0.6
Dispersion coefficient	3.86			
